# Adjuvant Transarterial Chemoembolization for Barcelona Clinic Liver Cancer Stage A Hepatocellular Carcinoma After Hepatectomy

**DOI:** 10.3389/fonc.2020.01754

**Published:** 2020-09-02

**Authors:** Yue-Lin Zhang, Chun-Hui Nie, Feng Chen, Tan-Yang Zhou, Guan-Hui Zhou, Tong-Yin Zhu, Sheng-Qun Chen, Xin-Hua Chen, Hong-Liang Wang, Bao-Quan Wang, Zi-Niu Yu, Li Jing, Zhi-Min He, Jun-Hui Sun

**Affiliations:** ^1^Hepatobiliary and Pancreatic Interventional Treatment Center, Division of Hepatobiliary and Pancreatic Surgery, The First Affiliated Hospital, Zhejiang University School of Medicine, Hangzhou, China; ^2^Zhejiang Clinical Research Center of Hepatobiliary and Pancreatic Diseases, Hangzhou, China; ^3^Zhejiang Provincial Research Center for Diagnosis and Treatment of Hepatobiliary Diseases, Hangzhou, China; ^4^Department of Radiology, The First Affiliated Hospital, Zhejiang University School of Medicine, Hangzhou, China

**Keywords:** hepatocellular carcinoma, hepatectomy, recurrence, survival, transarterial chemoembolization

## Abstract

**Introduction:**

The care for patients with hepatocellular carcinoma (HCC) is challenging. This study is to evaluate the effect of adjuvant transarterial chemoembolization (TACE) for Barcelona Clinic Liver Cancer (BCLC) stage A HCC patients after hepatectomy.

**Methods:**

Consecutive HCC patients with BCLC stage A, treated by hepatectomy alone (HA) or hepatectomy with TACE (HT), were retrospectively enrolled. Propensity score matching (PSM) was used to balance baseline differences. The recurrence-free survival (RFS) and overall survival (OS) were evaluated using the Kaplan-Meier. The impact of TACE on survival outcome was determined by Cox hazard regression.

**Results:**

After PSM, 230 patients (115 HT and 115 HA) were enrolled in the analysis. The 1-, 3-, and 5-year RFS rates were 87.0, 63.5, and 50.4%, respectively, for the HT group, and 87.8, 67.0, and 58.3% for the HA group. The OS rates at 1-, 3-, and 5-year were 99.1, 93.9, and 87%, respectively, for the HT group, and 100, 92.2, and 88.7% for the HA group. No significant differences were seen in either the RFS (log-rank test, χ^2^ = 0.891, *p* = 0.345) or OS (log-rank test, χ^2^ = 0.146, *p* = 0.702) between the specific pairs of two groups. Cox regression identified that TACE was not the factor affecting RFS or OS (*p* = 0.399; HR 0.847; 95% CI 0.576–1.245 for RFS vs. *p* = 0.989; HR 0.995; 95% CI 0.471–2.100 for OS).

**Conclusion:**

Our data indicate that TACE is not an effective intervention in the adjuvant setting for BCLC stage A HCC after hepatectomy.

## Introduction

Hepatocellular carcinoma (HCC) is one of the most frequently encountered malignancies globally, with the second-highest cancer-related mortality rate (1). Hepatectomy is the most widely practiced therapy, as it is a potentially curative treatment for HCC (2). However, long-term survival after hepatectomy is unsatisfactory because more than 70% tumor recur during the first 5 years (3). The prevention of tumor recurrences is the key to improve the outcome of liver resections.

The Barcelona Clinic Liver Cancer (BCLC) staging system including is widely used for HCC staging and treatment (4). Current clinical practice guidelines do not endorse any particular adjuvant therapy after hepatectomy but do recommend more and larger studies that undertake lower risks of systematic error (2, 4). Transcatheter arterial chemoembolization (TACE) has recently been reported as a postoperative adjuvant therapy for HCC patients. Previous clinical studies (5–7) showed that postoperative TACE significantly reduce tumor recurrence and improve the overall survival of patients with resectable BCLC stage B HCC or high recurrence risk (exceeding 5 cm in diameter or multiple tumors or vascular invasion) after curative liver resection. Nevertheless, none of these studies cover BCLC stage A HCC (single tumor or up to 3 tumors ≤3 cm), for which the BCLC staging system recommends surgical resection as the best option (8). The efficacy of TACE as adjuvant therapy after hepatectomy for patients with BCLC stage A HCC is not clear.

To further investigate the efficacy of TACE as adjuvant therapy after hepatectomy for patients with BCLC stage A HCC, we conducted a cohort study to follow up the survival outcome of BCLC stage A HCC who underwent hepatectomy alone or had postoperative adjuvant TACE.

## Materials and Methods

The study protocol was approved by the Ethics Committee of the First Affiliated Hospital, Zhejiang University School of Medicine, and the written informed consent was obtained from all the patients.

### Patients

In this retrospective cohort study, consecutive patients with BCLC stage A HCC, who underwent curative hepatectomy, were enrolled from January 2012 to August 2014 at the First Affiliated Hospital, Zhejiang University School of Medicine. The inclusion criteria: (1) the HCC diagnosis was confirmed by pathologic examination; (2) the patients had a stage A HCC using the BCLC staging system; and (3) histologic evidence of tumor-free margins on the resected tissues (defined as the distance between the cancer tissue and resected tissue margins is 1 cm or more). The exclusion criteria: (1) an intrahepatic recurrence within 2 months after curative hepatectomy; (2) the presence of other malignant tumors; and (3) loss of patients to follow-up.

Patients were divided into two groups: (1) HCC underwent hepatectomy with adjuvant TACE (HT group) and (2) HCC underwent hepatectomy alone (HA group). Standard demographic and clinical data potentially related to recurrence and survival were collected: gender, age, hepatitis, cirrhosis, tumor characteristics, surgical margin, and pathologic results.

### Adjuvant TACE

Postoperative adjuvant TACE was performed 4–6 weeks after hepatic resection, according to the Eastern Cooperative Oncology Group (ECOG) performance status and patient liver function. A 5-F angiographic catheter (Cook Inc., Bloomington, IN, United States) was introduced into the common hepatic artery through femoral artery, then hepatic angiography was performed to evaluate the arterial blood supply to the liver. 150 mg oxaliplatin (Hengrui Medicine Co., Ltd., Jiangsu, China) was slowly infused into proper hepatic artery, followed by an emulsion of 20 mg pirarubicin (Shenzhen Main Luck Pharmaceuticals Inc., Shenzhen, China) and 2–4 mL lipiodol (Lipiodol Ultrafluide, Guerbet, Aulnay-sous-Bois, France) using the microcatheter (Terumo, Tokyo, Japan). After 4–6 weeks, these patients underwent a complete assessment.

### Follow-Up

All patients were followed-up every 2 to 3 months during the first year and then every 3 to 6 months after surgery until death or dropout from the follow-up (4). On the follow-up visits patients tested ECOG, liver function, serum alpha-fetoprotein (AFP), abdominal ultrasonography, and CT or MRI scan. The primary endpoint for this study was recurrence-free survival (RFS), defined as the interval from surgery to the first recurrence. Secondary endpoints were overall survival (OS), defined as the interval from surgery to the date of death.

The diagnosis of tumor recurrence or metastasis was based on cytologic/histologic evidence or non-invasive diagnostic by the EASL (2). Two senior radiologists independently reviewed images. If any discrepancy in CT or MRI scans, the final diagnosis was made after reviewing all clinical information. After tumor recurrence was confirmed, the patients were treated according to the practice guidelines, which included curative treatments (surgical resection, liver transplantation, or radiofrequency ablation) and/or non-curative treatments (TACE, percutaneous ethanol injection, radiotherapy, or systemic therapy) to improve survival.

### Statistical Analysis

Sample sizes were computed using the RFS as the primary endpoint. Based on previous study, the 5-year RFS rate after curative resection was 40% (9). We expected a 5-year RFS rate in the adjuvant TACE group of 60%. Using a two-sided test with 80% power at a significance level of 5%, the minimum sample size in each group was estimated to be 94 patients.

To eliminate potential baseline confounding factors and isolate the effects of adjuvant TACE, propensity score matching (PSM) was used to balance baseline differences and thereby simulate random group allocation. The propensity score model included all variables known to be associated with survival outcomes. A one-to-one nearest-neighbor matching without replacement was used to match patients based on the logistic regression of the propensity score within a caliper of 0.05. That is to say, one patient from the HT group could get matched with one patient from the HA group with a similar propensity score.

Quantitative data were expressed as the mean ± standard deviation or median (range), as appropriate. Categorical data between HT and HA groups were compared using the chi-square or Fisher’s exact test, while quantitative data were compared using the Student’s *t*-test. Survival curves in this study were analyzed using the Kaplan-Meier to measure RFS and OS and compared using the log-rank test. Multivariable Cox regression analyses were used to identify the prognostic significance of the variables to predict RFS and OS. For subgroup analyses, multiple individual Cox models were used separately from treatment comparisons for each factor. Statistical analysis of the data was performed using SPSS V.25.0 (IBM, Armonk, NY, United States) and R V.3.6.1^1^ with the add-on packages survival, forestplot and survminer. A *p* < 0.05 was considered statistically significant, and all statistical tests were two-sided.

## Results

### Patients

**FIGURE 1:**
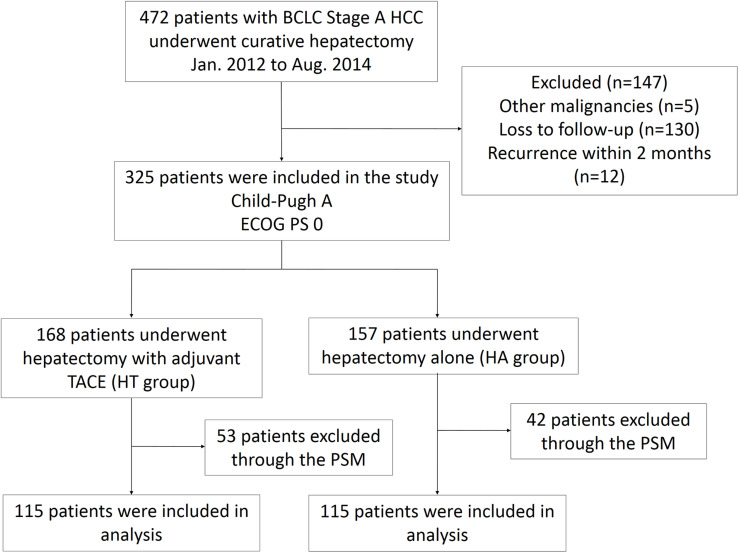
Flow diagram for extracting eligible cases for comparison. BCLC, Barcelona Clinic Liver Cancer; HCC, hepatocellular carcinoma; TACE, transarterial chemoembolization; PSM, propensity score matching; ECOG, Eastern Cooperative Oncology Group; PS, performance status.

From January 2012 to August 2014, 472 patients with BCLC stage A HCC underwent curative hepatectomy. Among them, 147 patients were excluded due to the presence of other malignancies (5 patients), loss to follow-up evaluations (130 patients), or HCC recurrences within 2 months (12 patients). Three hundred and twenty-five patients (157 HA patients and 168 HT patients) were included in the study. Then, 95 patients (42 HA patients and 53 HT patients) were excluded using one-to-one matching of the propensity scores to balance baseline differences between two groups. Finally, 230 patients (115 HA patients and 115 HT patients) were enrolled in the analysis. All patients had a good performance status and liver function (ECOG PS 0 and Child-Pugh A). The flow diagram demonstrating the screening and grouping of participants in the study is shown in Figure 1.

The baseline characteristics of the patients were well-matched between the two groups after PSM. The tumor characteristics, such as the tumor size (3.40 ± 2.00 for HA and 3.55 ± 1.61 for HT), tumor number, AFP level, presence of a tumor capsule, pathologic microvascular invasion, and histologic differentiation also were similar between the two groups (Table 1).

**TABLE 1:** Baseline demographic and disease characteristics.

Variable	Before propensity score matching			After propensity score matching		
	HT group (*n* = 168)	HA group (*n* = 157)	*P*-value	HT group (*n* = 115)	HA group (*n* = 115)	*P-*value
**Gender**						
Male	147	125	0.055	97	99	0.710
Female	21	32		18	16	
**Age, year**						
≤60	120	103	0.258	78	76	0.779
>60	48	54		37	39	
**HBsAg**						
Positive	149	132	0.224	99	97	0.710
Negative	19	25		16	18	
**HBeAg**						
Positive	41	42	0.628	28	27	0.877
Negative	127	115		87	88	
**HBV-DNA**						
Positive	71	57	0.272	44	44	1.000
Negative	97	100		71	71	
**Liver cirrhosis**						
Positive	118	123	0.095	82	88	0.368
Negative	50	34		33	27	
**Tumor size, cm**	4.11 ± 2.24	3.10 ± 1.86	<0.001	3.55 ± 1.61	3.40 ± 2.00	0.533
**Tumor number**						
One	147	150	0.010	107	108	0.789
Two or Three	21	7		8	7	
**Tumor capsule**						
Complete	64	75	0.078	47	48	0.893
Incomplete	104	82		68	67	
**Microvascular invasion**						
Yes	16	11	0.411	10	9	0.811
No	152	146		105	106	
**Histological differentiation**						
High and/or moderate	92	107	0.013	70	68	0.788
Low	76	50		45	47	
**Serum AFP, ng/mL**						
≤400	122	126	0.106	91	88	0.634
>400	46	31		24	27	
**Preoperative chemoembolization**						
Yes	17	13	0.567	8	8	1.000
No	151	144		107	107	
**Surgical margin, cm**						
≥2	109	110	0.319	79	84	0.468
<2	59	47		36	31	
**Operative blood loss, mL**						
≤500	146	148	0.024	105	107	0.623
>500	22	9		10	8	

*HT, hepatectomy with TACE; HA, hepatectomy alone; AFP, alpha-fetoprotein.*

### Survival

At the time of censor, 105 patients (45.7%) developed recurrence, 57 patients in the HT group, and 48 patients in the HA group, respectively. 28 patients (12.2%) had died, 15 patients in the HT group, and 13 patients in the HA group died of tumor-related causes.

**FIGURE 2:**
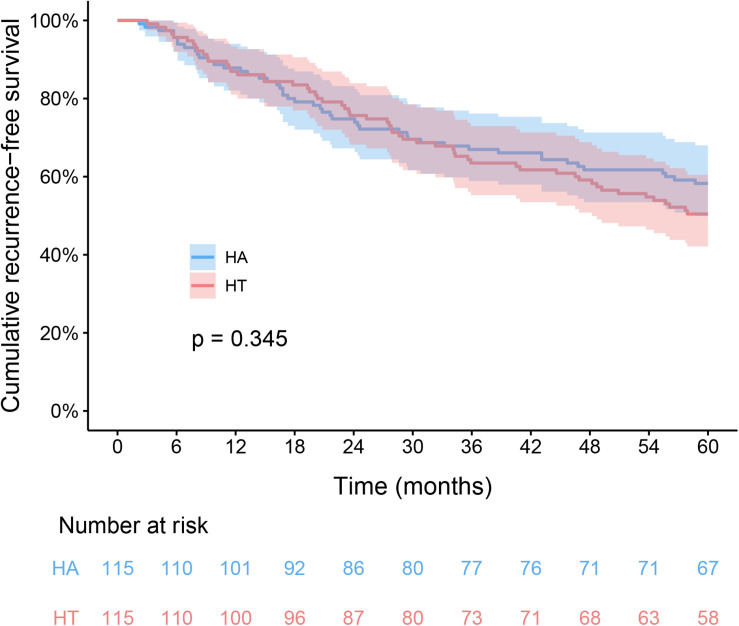
Kaplan-Meier analysis of recurrence-free survival between HT and HA groups. HT, hepatectomy with TACE; HA, hepatectomy alone.

**FIGURE 3:**
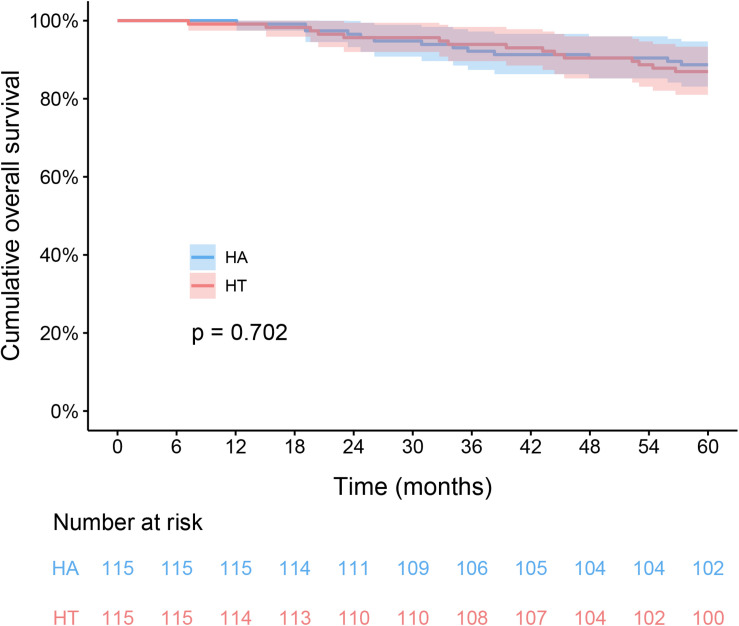
Kaplan-Meier analysis of overall survival between HT and HA groups. HT, hepatectomy with TACE; HA, hepatectomy alone.

In the HT group, the 1-, 3-, and 5-year RFS rates were 87.0, 63.5, and 50.4%, respectively. The corresponding figures for the HA group were 87.8, 67.0, and 58.3%, respectively. The RFS difference were not significantly in HT group compared with the HA group (log-rank test, χ^2^ = 0.891, *p* = 0.345, Figure 2). The respective 1-, 3-, and 5-year OS rates were 99.1, 93.9, and 87% for the HT group, and 100, 92.2, and 88.7% for the HA group. The OS rates were similar between the HT and HA groups (log-rank test, χ^2^ = 0.146, *p* = 0.702, Figure 3).

**FIGURE 4:**
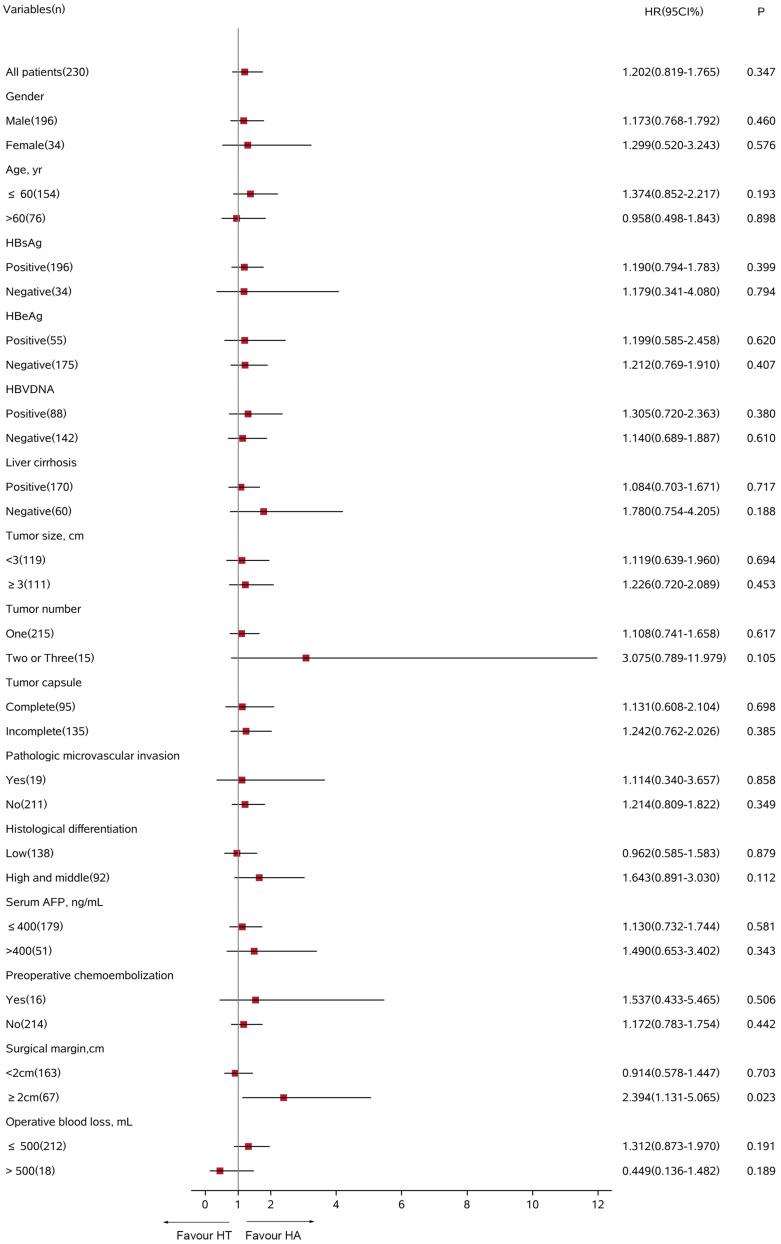
Subgroup analysis for recurrence-free survival between HT and HA groups using Cox regression analysis. HT, hepatectomy with TACE; HA, hepatectomy alone; CI, confidence interval; HR, hazard ratio; AFP, alpha-fetoprotein.

**FIGURE 5:**
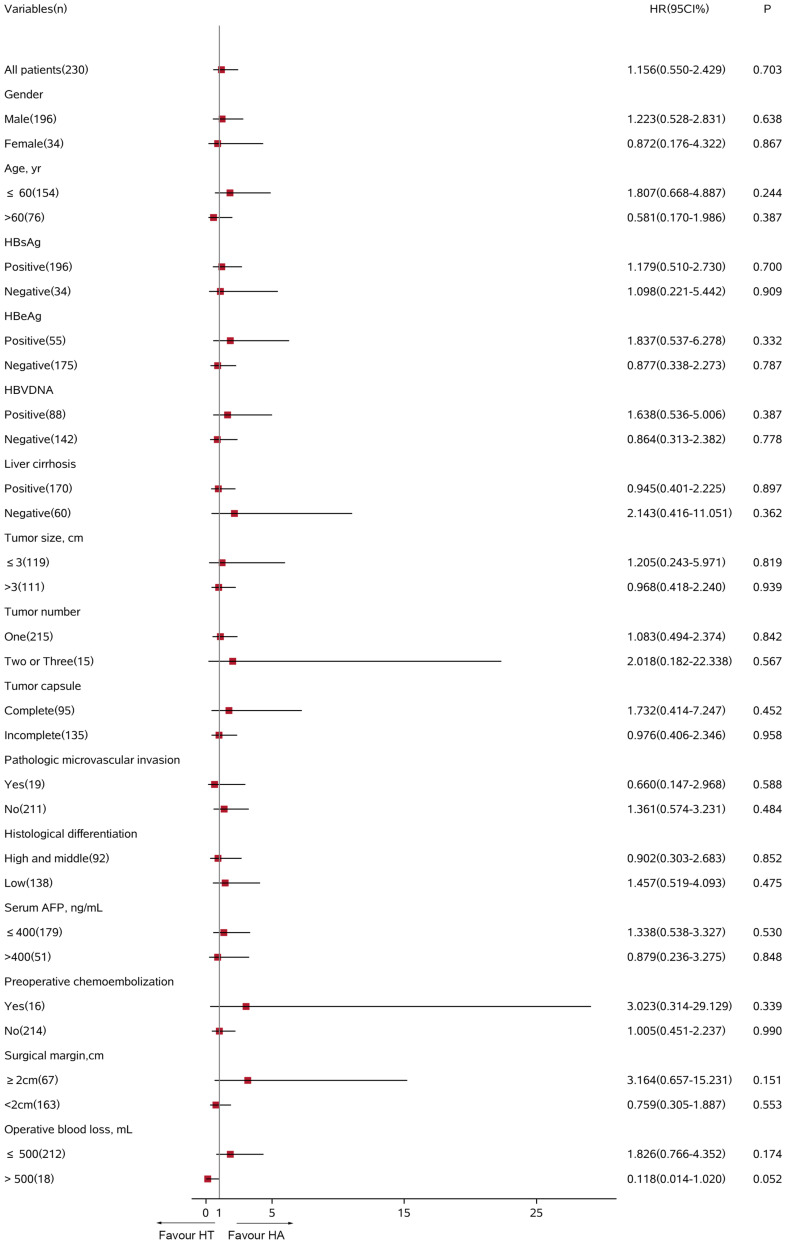
Subgroup analysis for overall survival between HT and HA groups using Cox regression analysis. HT, hepatectomy with TACE; HA, hepatectomy alone; CI, confidence interval; HR, hazard ratio; AFP, alpha-fetoprotein.

In the all-exploratory subgroup analyses, adjuvant TACE did not provide a clinical benefit for RFS [hazard ratio (HR) 1.202; 95% CI 0.819–1.765; *p* = 0.347]. Despite adjuvant TACE enhanced RFS for operative blood losses >500 ml, a significant difference was not detected in the HT group compared with that of the HA group (HR 0.449; 95% CI 0.136–1.482; *p* = 0.189, Figure 4). Similarly, in the OS subgroup analyses, a significant benefit from adjuvant TACE was not seen in patients with the following characteristics: ages >60 years (HR 0.581; 95% CI 0.170–1.986; *p* = 0.387) and operative blood losses >500 ml (HR 0.118; 95% CI 0.014–1.020; *p* = 0.052) (Figure 5).

Multivariate Cox regression analysis revealed that adjuvant TACE was not the impact factor for RFS or OS (*p* = 0.399; HR 0.847; 95%CI 0.576–1.245 for RFS vs. *p* = 0.989; HR 0.995; 95% CI 0.471–2.100 for OS). Tumor size and the presence of microvascular invasion were shown to be significant OS factors (Table 2).

**TABLE 2:** Uni- and multivariate analyses of recurrence-free survival (RFS) and overall survival (OS).

Variable	Univariate analysis		Multivariate analysis	
	χ^2^ value (Log-rank)	*P*-value	HR (95% CI)	*P*-value
**RFS**				
Treatment (HT vs. HA)	0.891	0.345	0.847 (0.576–1.245)	0.399
Gender (female vs. male)	3.061	0.080	0.703 (0.426–1.162)	0.169
HBsAg (positive vs. negative)	3.357	0.067	0.601 (0.312–1.160)	0.129
Tumor number (two or three vs. one)	3.083	0.079	0.603 (0.311–1.168)	0.134
Pathologic microvascular invasion (yes vs. no)	3.217	0.073	0.587 (0.313–1.101)	0.097
**OS**				
Treatment (HT vs. HA)	0.146	0.702	0.995 (0.471–2.100)	0.989
HBeAg (positive vs. negative)	4.181	0.041	0.471 (0.220–1.009)	0.053
Tumor size, cm (>3 vs. ≤ 3)	11.698	0.001	0.320 (0.122–0.834)	0.020
Microvascular invasion (yes vs. no)	15.492	<0.001	0.299 (0.123–0.725)	0.008
Operative blood loss, mL (≤500 vs. >500)	8.827	0.003	0.416 (0.163–1.059)	0.066

*HT, hepatectomy with TACE; HA, hepatectomy alone.*

## Discussion

This study include a large patient cohort. One group received hepatectomy with adjuvant TACE and the other received hepatectomy alone. The result revealed that postoperative adjuvant TACE does not reduce recurrence or improve OS. Although the role of postoperative adjuvant TACE was evaluated to improve the outcomes of resected HCC in several studies, the results have been controversial. Difference in patient selection made the controversial results. A randomized controlled trial revealed that adjuvant TACE after hepatectomy markedly improved the survival outcome of Stage III A HCC patients, including a high proportion of patients with macrovascular invasion (7). However, another prospective, randomized-controlled trial failed to display a significant difference in survival between the two groups, although the main aim of this study was to look into the effect of the dose in the prevention of tumor recurrence (10). Sun et al. (11) retrospectively reported that postoperative adjuvant TACE could prolong the survival of patients with microvascular invasion with 5-year survival rates that increased to 54.0%. However, our study focused on BCLC stage A HCC (single tumor or up to 3 tumors ≤3 cm). Thus, the histopathologic factors of HCC in our study were significantly different from previous studies.

The rationale of adjuvant TACE after curative hepatectomy was to prevent intrahepatic recurrence by killing residual microscopic tumor cells in the remnant live. It can also eliminate tumor cells that might have been shed from tumor masses removed during liver surgery. Our negative results show that adjuvant TACE did not improve the outcomes of BCLC stage A HCC patients are likely related to the following factors. First, immune surveillance to control tumor recurrences and metastases could be responsible. Adjuvant TACE might have depressed host immunity against tumor progression and affected hepatocyte regeneration, resulting in poor overall or recurrence-free survival (12). Second, the recurrent tumors usually have different clones compared with those of the malignant primary tumors (13). By eliminating the subpopulation of drug-sensitive tumor cells, chemotherapy accelerate the formation of new clonal variants from the surviving subpopulation and allow cells to proliferate with higher metastatic capabilities (14).

This study revealed that tumor size and microvascular invasion were both independent prognostic factors of OS, similar to the results of previous studies (15, 16). A large tumor burden is closely associated with increased invasiveness, which was reflected in a higher incidence of microvascular invasion and poor survival (17, 18). Microvascular invasion is present in 20% of tumors 2 cm in size, 30–60% of nodules 2–5 cm in size, and up to 60–90% of nodules larger than 5 cm (19). The proportion of HCC cases with microvascular invasion was only 8.3% in our study, resulting in good long-term survival rates.

There are potential limitations of this study. First, it was a retrospective study with all of the inherent defects of these types of studies and is likely subject to subtle selection biases, even after PSM. Second, this is a single-center study, and the outcome is not the same as patients with BCLC stage A HCC in other countries because of demographics and the underlying causes. The multi-center randomized controlled trials involving patients with BCLC stage A HCC should examine in more detail the effects of postoperative TACE.

## Conclusion

Transarterial chemoembolization as adjuvant treatment after hepatectomy for BCLC stage A HCC did not reduce tumor recurrences or improve the overall survival. The adjuvant setting remains an area of high unmet need in HCC management, and further research into strategies to prevent BCLC stage A HCC recurrence is needed.

## Data Availability Statement

All datasets presented in this study are included in the article/supplementary material.

## Ethics Statement

The studies involving human participants were reviewed and approved by the Ethics Committee of the First Affiliated Hospital, Zhejiang University School of Medicine. The patients/participants provided their written informed consent to participate in this study.

## Author Contributions

Y-LZ and J-HS contributed to conception and design. Y-LZ, C-HN, and FC contributed to development of methodology (provided, acquired, and managed patients, etc.). Y-LZ, T-YaZ, and G-HZ contributed to analysis and interpretation of data (e.g., statistical analysis and computational analysis). Y-LZ, T-YiZ, S-QC, X-HC, H-LW, and J-HS contributed to writing, review, and/or revision of the manuscript. Y-LZ, B-QW, Z-NY, LJ, and Z-MH contributed administrative, technical, or material support (i.e., reporting or organizing the data and constructing databases). All authors contributed to the article and approved the submitted version.

## Conflict of Interest

The authors declare that the research was conducted in the absence of any commercial or financial relationships that could be construed as a potential conflict of interest.

## Footnotes

1 https://www.r-project.org
